# Exploring the Potential of Mitochondria-Targeted Drug Delivery for Enhanced Breast Cancer Therapy

**DOI:** 10.1155/ijbc/3013009

**Published:** 2025-03-03

**Authors:** Yalda Ghazizadeh, Seyedeh Elnaz Sharifi-Ardani, Negin Tajik, Roya Mirzaei, Jalal Pourahmad

**Affiliations:** ^1^Student Research Committee, School of Pharmacy, Shahid Beheshti University of Medical Sciences, Tehran, Iran; ^2^Pharmaceutical Sciences Research Center, Shahid Beheshti University of Medical Sciences, Tehran, Iran; ^3^Department of Toxicology and Pharmacology, School of Pharmacy, Shahid Beheshti University of Medical Sciences, Tehran, Iran

**Keywords:** breast cancer, immunological properties, mitochondria, nanotechnology

## Abstract

Breast cancer stands as the utmost prevalent malignancy in women, impacting the epithelial tissue of the breast and often displaying resistance to effective treatment due to its diverse molecular and histological features. Current treatment modalities may exhibit decreasing efficacy over time and can lead to disease progression. The mitochondria, a crucial organelle responsible for cellular metabolism and energy supply, stand highly sensitive to both heat and reactive oxygen species, presenting an assuring target for photodynamic and photothermal therapies (PTTs) in cancer cure. The employment of nanodrug carriers for combination deliveries holds promise in addressing challenges related to drug degradation and off-target toxicity. By circumventing the reticuloendothelial system, nanocarriers bolster the drug's bioavailability at the intended site and ensure controlled codelivery of multiple drugs, thereby maintaining the normal pharmacokinetic features and the regular pharmacodynamic characteristics of different therapeutic mechanisms. The precision and efficacy of this innovative technology have revolutionized drug delivery, substantially enhancing treatment effectiveness. In the pursuit of targeting mitochondrial modifications in cancer cells, various combination therapies such as photodynamic therapy (PDT), PTT, and chemodynamic therapy (CDT) have been explored. These therapies have improved the efficiency of mitochondria-targeted cancer treatment due to their advantageous properties of minimal toxicity, noninvasiveness, reduced drug resistance, and a safer profile. Our review article provides an exhaustive overview of alterations in the mitochondrial environment in BC, their impact on BC development, potential mitochondrial targets for BC treatment, nanotherapeutic approaches for targeting mitochondria, and the limitations of these approaches.

## 1. Introduction

Breast cancer, also known as BC, is a prevalent form of cancer that primarily affects women [1]. The malignant growth develops in the epithelial tissues of the breast and is the primary cause of cancer-related fatalities in women [2]. Several factors contribute to the development of BC, such as exposure to external and internal estrogens, an unhealthy lifestyle, obesity, excessive alcohol consumption, and harmful environmental aspects. Genetic factors account for approximately 15% of BC cases. Mutations in the BRCA1 and BRCA2 genes, which are associated with BC, have been extensively researched [3]. The genes in question are instrumental in the process of DNA repair. Their deactivation can potentially cause chromosomal instability, thereby contributing to the onset of various forms of cancer [4]. The highly diverse characteristics of BC, encompassing a broad spectrum of molecular features and histological profiles, present a substantial challenge to achieving effective treatment [5]. Various treatment prospects are obtainable for BC based on the stage and type of the condition. These options contain chemotherapy, immunotherapy, gene therapy, hormone therapy, and surgery. It is important to note that chemotherapy is the most frequently used approach [6]. Chemotherapy drugs that are small molecules cannot specifically target tumors, leading to harmful side effects and significant toxicity on normal tissues [7–9]. Long-term chemotherapeutic treatment can elevate the metastasis and invasion of cancer cells [10].

Targeted therapies such as estrogen receptor (ER)–targeted and HER2-targeted treatments are highly detailed to certain subtypes and have been shown to be extremely effective as first-line therapy [11, 12].

As treatment progresses and the disease advances, the efficacy of these treatments may decrease over time [12]. The majority of patients detected with triple-negative breast cancer (TNBC), described as being negative for HER2, ER, and PR, typically undergo treatment involving a combination of radiotherapy and chemotherapy or just chemotherapy alone. The treatment of TNBC poses several challenges, including the diverse nature of the disease, high rates of metastasis, uncertain prognosis, and the absence of specific targeted therapies [13, 14]. Hormonal therapy is a commonly employed approach for treating BC in patients with ER-positive tumors; however, it often causes the expansion of drug resistance [15]. Mitochondria represent a very promising method for precision cancer therapy. Mitochondria play a critical role in energy regulation and the progression of cancer, rendering them a vital focal point in cancer research. The metabolic shift witnessed in cancer, chiefly the Warburg effect, underscores the essential involvement of mitochondria in cancerous cells [16, 17].

Lipid-based nanoparticles possess unique immunological properties that can be effectively utilized to bolster the antitumor immune response. By strategically incorporating immunostimulatory molecules or antigens into the nanoparticle formulation, researchers can effectively stimulate the activation of crucial immune cells, such as cytotoxic T cells and NK (natural killer) cells. These immune cells are instrumental in the process of eliminating cancer cells. The resultant immune-stimulating effect can work synergistically with mitochondria-targeted therapy, thereby offering the potential to significantly enhance treatment outcomes for cancer patients [18, 19].

Utilizing nanodrug carriers for combination deliveries is an exceptionally effective approach to address these challenges. Nanocarriers significantly enhance the drug's bioavailability at the targeted site while effectively reducing drug degradation via bypassing the RES (reticuloendothelial system). The use of nanocarriers also allows for the minimization of off-target toxicity. Additionally, the delivery systems of nanodrugs enable the precise codelivery of multiple drugs while preserving the normal pharmacokinetics features and the regular pharmacodynamics characteristics of different therapeutic agents. This groundbreaking technology revolutionizes drug delivery, providing unparalleled precision and efficiency, ultimately leading to vastly improved treatment effectiveness [6, 20–22].

The drawbacks associated with mitochondrial-targeted therapies in BC warrant consideration. Despite their initial promising results, cancer cells may develop resistance to therapies targeting mitochondrial functions through mechanisms such as alterations in mitochondrial activity or the activation of compensatory survival pathways [17, 23]. This phenomenon can significantly diminish the effectiveness of these therapies over time. Furthermore, certain mitochondrial-targeted therapies may unintentionally harm healthy cells, leading to adverse side effects such as nausea, fatigue, and potential organ damage. These off-target effects may limit the use of these therapies in specific patient populations or necessitate careful dosing and monitoring to mitigate associated risks [24, 25].

The following review is dedicated to examining the modifications observed in mitochondria within tumor microenvironments, their influence on the expansion of BC, potential targets within the mitochondria for treating BC, nanotherapeutic strategies that specifically target mitochondria, and the associated constraints of these methodologies.

## 2. Mitochondria Alterations in Cancer

The mitochondria are dynamic organelles characterized by a dual-membrane structure, consisting of an outer mitochondrial membrane (OMM) and an inner mitochondrial membrane (IMM) divided by an intermembrane space. They undergo continual fusion (assembly) and fission (division), resulting in a state of constant flux rather than static structures [26, 27]. The function and structure of mitochondria in tumor cells vary significantly from those in normal cells. Tumor cells have adapted their metabolism to enhance their chances of survival, regardless of nutrient availability. These alterations are attributed to the Warburg effect, which is named after Otto Warburg, the scientist who first described it a century ago [28]. Warburg's research revealed that cancer cells have the capability to uptake glucose and convert it into lactate even in the presence of oxygen. He postulated that this phenomenon stemmed from defects in mitochondrial catabolism, leading to aerobic glycolysis in cancer cells. The extensive metabolic reprogramming in cancer cells significantly influences their catabolic processes, including autophagy, necrosis, and apoptosis [28–30]. Enlarged and fragmented mitochondria are frequently observed in cancer cells, with tubular mitochondria primarily seen during the S phase and uniformly damaged mitochondria prevalent in the M phase. Additionally, cancer cells exhibit a notably more hyperpolarized mitochondrial membrane potential (MMP) compared to normal cells [31]. Epithelial breast tumor cells demonstrate an elevated expression of OXPHOS, facilitating increased ATP production. In a study involving MCF-7 cells, it was observed that oxidative metabolism contributes to 80% of ATP generation. It is important to note that heightened glucose consumption does not invariably result in an escalation of glycolysis rates [32]. There is growing evidence suggesting that the microenvironment within tumors exhibits a dysregulated expression of mitochondrial proteins, specifically fusion proteins, fission proteins, and proteases. BC cells, in particular, have been shown to overexpress certain mitochondrial GTPases, such as dynamin-related protein 1 and Mfn1/2, which are known to be critical components in the processes of cell division and mitochondrial fusion [31]. Excessive generation of reactive oxygen species (ROS) within cancer cells can result in the oxidation of various cellular macromolecules, such as DNA, lipids, and proteins. This heightened level of ROS within the cytosol triggers a series of oxidative signaling pathways, ultimately leading to the activation of specific transcription factors, namely, NRF2, AP1, NF-*κ*B, and p53. These transcription factors are known to play a crucial role in regulating gene expression that is associated with tumor genesis [33].

BC cells experience genetic changes due to oxidative stress caused by estrogen, leading to the generation of ROS. Additionally, the suppression of ERs results in an adaptation to oxidative stress, ultimately promoting more aggressive growth potential [34]. When cells are deprived of oxygen (hypoxia), the electron transport chain induces ROS, which are then released into the cytosol. This release leads to oxidative stress and activates associated signaling pathways. The intermediate metabolites of the TCA cycle play a critical role in tumor development. In cancer cells, dysfunctional mitochondria not only hinder TCA cycle enzymes but also significantly contribute to lipid synthesis by using glutamine for reductive carboxylation, thereby providing essential energy. Mutations in genes encoding TCA cycle enzymes can profoundly impact the activation of hypoxia-inducible factors (HIFs) [33]. There is compelling evidence indicating that autophagy is pivotal in the metabolic flexibility of cancer cells. By breaking down organelles, lipids, and proteins, this process not only generates fresh metabolic substrates but also ensures that cells can meet their bioenergetic requirements. Tumor progression has been unequivocally associated with mitophagy, the process of degrading dysfunctional and obsolete mitochondria [34].

**Figure 1:**
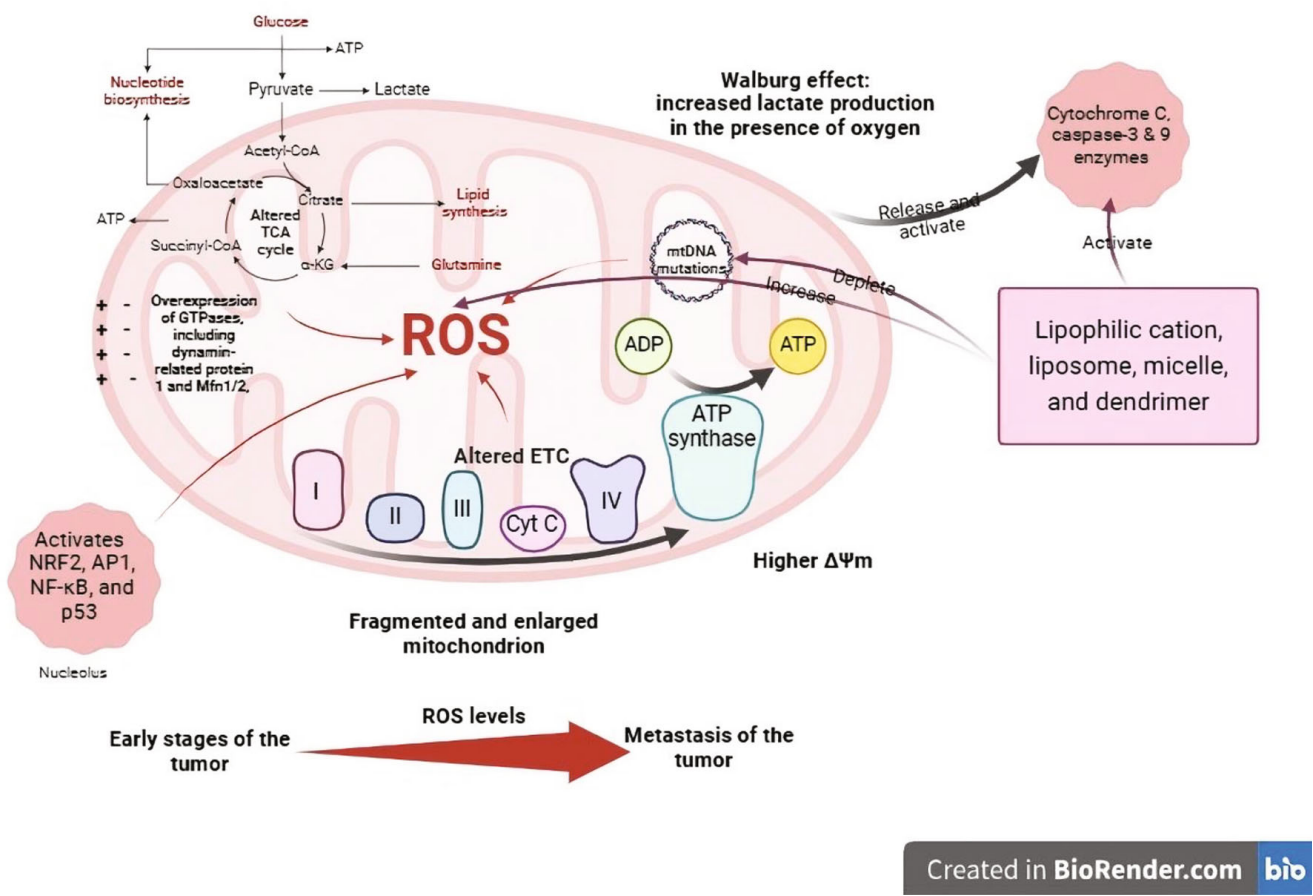
Mitochondrial alterations in cancer cells. The Warburg effect, which causes increased lactate generation in cancer cells even in the presence of oxygen, is a hallmark of mitochondrial failure. Aside from this, a number of additional changes are noticeable. These include disturbances to the TCA cycle and ETC dynamics. Furthermore, the activation of genes such as NF-*κ*B, NRF2, AP1, and P53, as well as the overexpression of GTPases such as Mfn1/2 and dynamin-related protein 1, contribute to mitochondrial dysfunction. These collective modifications eventually lead to increased reactive oxygen species (ROS) generation, highlighting the complex connection between mitochondrial abnormalities and cancer growth. Furthermore, cancer cell mitochondria show enhanced permeability and morphological changes such as enlargement and fragmentation. Lipophilic cations, liposomes, micelles, and dendrimers discussed in the text lead to increased production of reactive oxygen species (ROS), depletion of mitochondrial DNA, increased permeability of the mitochondrial membrane, activation of Bax, suppression of Bcl-2, release of cytochrome C, and subsequent activation of Caspase 3 and Caspase 9 enzymes, which ultimately results in apoptosis (created in http://BioRender.com).

The process of apoptosis represents a meticulously regulated form of programmed cell death encompassing intrinsic and extrinsic pathways. Various cellular stressors initiate the intrinsic pathway, whereby Bcl-2 proteins effectively regulate the release of specific caspase activators within the mitochondria. The p53 protein or AKT functions as a robust damage sensor, activating BH3 proteins to initiate proapoptotic molecules such as Bad, Bax, Bid, and Bak while counteracting the inhibitory effects of antiapoptotic molecules like Mcl-1, Bcl-XL, and Bcl-2. Recent research strongly suggests that numerous anticancer medications induce apoptosis throughout the intrinsic pathway [31]. The impairment of the apoptotic process can lead to the proliferation of genetically altered cells and the onset of cancer. Antiapoptotic proteins like Bcl-XL and Bcl-2 are intricately associated with the initiation, advancement, and resistance to the treatment of neoplasms [33]. For more information and a visual representation, please refer to Figure 1, which presents a comprehensive overview of the key concepts and findings discussed in this review.

## 3. Targeting Mitochondria for Cancer Therapy

Targeting mitochondria-related mechanisms is a highly effective strategy in combating tumor cells, especially during the early stages of tumor development when apoptotic mechanisms are not functioning properly [33]. Various strategies are employed to target mitochondrial abnormalities in cancer cells. Cancer cells demonstrate a more heightened mitochondrial membrane potential (*ΔΨ*m) compared to normal cells. Exploiting the lower transmembrane potential of normal cells relative to cancer cells authorizes the design of small molecules that selectively target and accumulate inward the mitochondria of cancer cells. To access the mitochondrial matrix, two critical prerequisites are necessary: possessing a delocalized positive charge and demonstrating lipophilicity. Several compounds possess these properties, including Rh123, MKT-077, and F16. Notably, MKT-077 can deplete mtDNA while effectively inhibiting Complexes III and IV. Targeting OXPHOS agents is a highly effective strategy for combating the advancement of BC. Derivatives of benzoquinones, monoamine-oxidase inhibitors, and analogs of vitamin E possess the ability to effectively inhibit Complex II due to their similarity to ubiquinone. AS2O3 and resveratrol can effectively inhibit Complex I, while benzoquinone derivatives, MitoVES, or *α*-tocopheryl succinate (*α*-TOS) can inhibit Complex II. These mechanisms can cause mitochondrial malfunction in tumor cells, ultimately leading to cell death. Another promising strategy to slow down cancer progression is to target the Bcl-2 protein family and HK2 within tumor cells. Gossypol can effectively inhibit Bcl-2 and Bcl-XL, while simultaneously increasing the levels of Mcl-1.3-bromopyruvate, lonidamine, and metformin can trigger apoptosis by detaching HK2 from mitochondria and hindering glycolysis. Additionally, targeting mtDNA with molecules like vitamin K3, ditercalinium, cisplatin, and etoposide has shown promise in effectively combating cancer [35].

The membrane potential of the IMM and OMM can indeed be modified by specific cancer medications, such as cisplatin, paclitaxel, and arsenic trioxide. Furthermore, other cancer treatments, including lipophilic cations and BH3 mimics, are capable of disrupting membrane potential by inducing oxidative stress in the mitochondria. Numerous well-established studies have unequivocally shown that inducers of ROS effectively prompt mitochondrial-dependent apoptosis in cancer cells by elevating ROS levels within these cells. Some prominent examples of these ROS inducers encompass rapamycin, resveratrol, cisplatin, DOX, sorafenib, and 5-fluorouracil (5-FU) [29]. Combination therapies like PDT, PTT, and CDT significantly improve the effectiveness of mitochondria-targeted cancer treatment due to their superior safety profile, minimal toxicity, noninvasive nature, and reduced drug resistance. PDT uses photosensitizers (PSs), light, and oxygen to eliminate tumors. PTT destroys tumor cells through thermal damage using near-infrared (NIR) light and a potent photothermal agent. CDT involves a Fenton or Fenton-like reaction to target excessive intracellular hydrogen peroxide (H_2_O_2_) within tumor tissues, resulting in the production of potent hydroxyl radicals (•OH) [31]. However, the FDA has approved only a limited number of drugs that specifically target mitochondria [36].

## 4. Nanotherapeutic Approaches

Nanocarriers are new drug delivery systems that can cross biological barriers, protect bioactive agents, and enhance drug effectiveness. They come in various forms, such as polymeric nanoparticles, natural polymer conjugates, synthetic polymer conjugates, dendrimers, nanotubes, lipid-based carriers, carbon, titanium oxide, gold, and platinum nanoparticles. They can encapsulate therapeutic compounds. Researchers are studying their potential therapeutic applications [37]. In Table 1, refer to access to a more extensive set of detailed information.

### 4.1. Lipophilic Cations

The membrane potential of mitochondria is consistently highly negative, typically ranging from −160 to −180 mV. This indicates that positively charged molecules can effectively target mitochondria by leveraging electrostatic interactions. Furthermore, for cationic molecules to efficiently traverse the phospholipid bilayer of the IMM, they must possess an appropriate level of lipophilicity. Several small molecules with these properties have already been identified and are widely recognized as delocalized lipophilic cations (DLCs). Triphenylphosphine (TPP) was the pioneering small molecule used to specifically target mitochondria, owing to its exceptional lipophilicity and unique cationic nature [44]. The anticancer agent Dox is widely used and forms a conjugate with TPP. The amine group of Dox and the carboxyl group of TPP interact, leading to conjugation. A study demonstrated that the TPP-Dox compound was absorbed by BC cells treated with Dox to a greater extent than by untreated cells, which exhibited relatively lower absorption. These findings indicate that the TPP-Dox combination effectively overcame drug resistance in the cell line. However, the investigation also revealed that the compound's limited solubility may present challenges for in vivo models [45].

Rackham et al. discovered that the water-soluble ligand 1,3-bis (di-2-pyridylphosphino) propane (d2pypp) shows promising anticancer properties when combined with bischelated Au(I) bidentate phosphine. This combination has been found to reduce MMP, deplete glutathione pools, trigger the activation of Caspase 3 and Caspase 9, and ultimately lead to apoptosis in BC cells [46]. The study conclusively established the significant cytotoxic effects of five decyl polyhydroxy benzoates linked with TPP+ on all BC cell lines. These compounds not only induced sensitivity to the cytotoxic effects but also led to a decrease in mitochondrial transmembrane potential. Their ability to weakly uncouple OXPHOS triggered the mechanism responsible for cytotoxicity, demonstrating a broader therapeutic range and enhanced safety compared to other alternatives [47]. Four meso-tetraphenylporphyrin derivatives were developed in a recent study, each containing either triphenylphosphonium ions (P1 and P2) or triethylammonium ions (P3 and P4). Microscopic analysis indicated that all four derivatives target mitochondria. The study's results unveiled that P1–P4 demonstrated phototoxicity towards the BC cell line while exhibiting minimal dark toxicity at the concentrations tested [48]. Olelewe et al. have introduced AuPhos-19, a notable small molecule containing a chiral phosphine ligand, with the unique ability to selectively modulate mitochondrial metabolism. The lipophilic and cationic properties of AuPhos-19 render it an ideal candidate for engaging with mitochondrial OXPHOS. This compound effectively impedes mitochondrial respiration while also activating AMPK. The resultant disruption of mitochondrial function is evidenced by the depolarization of the mitochondrial membrane, the accumulation of ROS within the mitochondria, and the depletion of mitochondrial DNA attributed to AuPhos-19 [49]. A recent study has introduced a series of lipophilic, cationic Au(I) complexes of N-heterocyclic carbenes (NHCs). These complexes demonstrate a specific tendency to accumulate in mitochondria and effectively inhibit thioredoxin-reductase. Furthermore, they have been found to induce apoptosis while concurrently impeding the activity of thioredoxin-reductase [50].

### 4.2. Liposome

Liposome-based systems possess the capability to effectively target mitochondria and combat cancer due to their spherical shape and core of aqueous solution. Furthermore, their versatility and lack of immunological response make liposomes a promising option. They can be tailored into various morphologies, enclosed, composed of different components, and offer protection to therapeutic proteins [51, 62]. Biswas et al. addressed STPP-L's nonspecific cytotoxicity by synthesizing TPP-PEG-PE (PEG-PE with distal TPP attached to the PEG block) to reduce TPP's nonspecific cytotoxicity. They demonstrated that the conjugation of PTX with PEG-PE-TPP liposomes (TPP-PEG-PE-PTX) exhibited significantly greater cytotoxicity and antitumor activity than PTX-encapsulated unmodified liposomes (PL-PTX) [52, 63]. A different study employed a dendritic peptide moiety based on guanidinium as a liposomal delivery platform that emulated mitochondrial transmembrane signaling. Owing to its charge-reversible and pH-sensitive properties, this platform was found to augment tumor accumulation and cell penetration by selectively targeting the mitochondrion [16].

A study developed a folate receptor-targeted PEGylated liposome containing bioactive compounds from *Kappaphycus alvarezii* in order to increase the anticancer activity of the liposomes. PEGylated liposomes selectively impacted the MCF-7 cells' mitochondria, leading to an increase in ROS and possible disruption of the mitochondrial transmembrane potential [6]. BTP, which stands for 5,10,15,20-tetrakis (benzo[b]thiophene) porphyrin, is an innovative hydrophobic PS. A study has confirmed that the production of ROS in liposomal BTP-treated MCF-7 cells increases in a concentration-dependent fashion following irradiation. All liposomal BTPs successfully induced mitochondrial permeability transition, resulting in an enhanced permeability of the mitochondrial membrane, along with the activation of Caspases 3 and 7 [7]. Katopodi et al. developed a multipurpose nanostructured liposome to deliver celecoxib (CEL) and doxorubicin hydrochloride [77] simultaneously for BC that is resistant to doxorubicin. The MTS-R8H3 peptide entity on liposome surfaces in this study enhanced mitochondrial targeting and effectively produced ROS [8]. Liposomes conjugated with D-a-tocopheryl polyethylene glycol (PEG) 1000 succinate-TPP conjugate (TPGS1000-TPP) encapsulated with sunitinib and vinorelbine separately, as well as in combination, were specifically delivered into invasive BC cells, where they were actively taken up and concentrated within the mitochondria of the cells. This targeted approach enabled the liposomes to cause an immediate cytotoxic effect and trigger programmed cell death [56]. A study synthesized a GSH-sensitive artesunate conjugate (TPP-SS-ATS) and prepared TPP-SS-ATS-LS liposomes. The cytotoxicity was significantly enhanced, leading to an increase in the inhibition rate of tumor growth from 37.7% to 56.4%. Furthermore, BC cells have been found to exhibit mitochondrial dysfunction, leading to a decrease in ATP production and respiratory capacity [9, 57]. KLA-5-FU/PTX liposomes, which are liposomes modified with KLA and loaded with 5-FU and paclitaxel exhibited amplified cytotoxicity, enhanced drug transportation to mitochondria, and triggered apoptosis [10]. Liposomes containing berberine exhibited remarkable ABC transporter inhibition capabilities, as well as the ability to selectively accumulate in mitochondria. This accumulation led to the activation of the protein Bax and the suppression of Bcl-2 [59]. Topotecan-loaded liposomes, as well as a combination of daunorubicin and quinacrine or SS-02-Doxil, were effectively targeted to the mitochondria, resulting in an increased concentration of the drugs in this organelle. This caused the release of cytochrome C and activation of Caspases 3 and 9 enzymes, eventually triggering apoptosis [60–62]. A study delved into the efficacy and functional mechanism of epirubicin liposomes against BC cells. The findings indicated that these liposomes stimulated the activation of apoptotic enzymes such as Caspases 8, 9, and 3, as well as the upregulation of the proapoptotic protein Bax while suppressing the antiapoptotic protein Mcl-1. Additionally, the liposomes triggered a sequence of events that resulted in an increase in the production of ROS, ultimately leading to apoptosis [63]. In another study by Hu et al., liposomes carrying nanostructured dihydroartemisinin and epirubicin were developed. It has been discovered that the simultaneous administration of dihydroartemisinin and epirubicin effectively suppresses the function of Bcl-2, promotes the release of Beclin-1, and leads to the activation of Bax. Moreover, Bax triggered apoptosis, leading to the programmed cell death Type I in BC cells, whereas Beclin-1 instigated an intensified autophagy process, ultimately resulting in programmed cell death Type II in BC cells. Furthermore, these liposomes demonstrated an extended duration of drug circulation [11].

### 4.3. Micelle

Micelles are minute colloidal structures, typically measuring between 5 and 100 nm in particle diameter. They consist of molecules possessing dual water affinities. The formation of micelles necessitates specific temperature and concentration conditions for the amphiphilic molecules [64]. Polymeric micelles have the capacity to accumulate in the tumor microenvironment due to their nanoscale size, which enhances their permeability and retention [66]. This property allows lipophilic drugs to be transported within the core of the micelles. Widely utilized in therapeutic applications, polymeric micelles offer improved solubility and enhanced intestinal permeability. Their notable features include prolonged circulation and exceptional tumor accumulation, making them a valuable tool for tumor targeting in the field of therapeutics [65]. The combination of hydrophilic heptamethine cyanine dyes, IR780, and fluorescent dye Ce6 has led to the development of a promising sonosensitizer, PEGIR780@Ce6, by Han et al. This sonosensitizer not only shows improved absorption by tumor cells but also demonstrates the ability to efficiently generate ^1^O_2_, H_2_O_2_, and OH free radicals. Moreover, the tagging of PEGIR@Ce6 with IR780 enhances its potential for specifically targeting mitochondria in cancer cells. Additionally, the newly designed sonosensitizer exhibits prolonged persistence in cells compared to IR780 and free Ce6 alone. The in vivo and in vitro studies have clearly highlighted the potential of this novel sonosensitizer in suppressing the invasion and migration of breast tumor cells [67]. Zhang et al. have made a significant breakthrough by synthesizing a novel PS that specifically targets mitochondria. This PS, based on IR780 iodide and a heat shock protein 90 inhibitor called BIIB021, shows great promise. By selectively accumulating in mitochondria and releasing BIIB021 upon NIR irradiation, it effectively reduces the heat tolerance of cells. Consequently, there is a decrease in the MMP, leading to rapid effects on critical factors involved in intrinsic apoptosis, such as Cyt-C, Bcl-2, Bax, and Caspase 9. This innovative PS has demonstrated remarkable effectiveness in achieving mitochondrial photothermal therapy (MT-PTT) [68]. The study introduced a novel approach by developing micelles loaded with DOX and EVO, which demonstrated promising potential for targeting metastatic BC cells. These micelles utilized triphenylphosphonium cations to specifically target the mitochondria, and the subsequent breakdown of disulfide bonds by GSH effectively facilitated the release of DOX and EVO in close proximity to the mitochondria. The resulting damage to the mitochondrial membrane by EVO allowed a significant amount of DOX to enter the mitochondria, thereby enhancing the antitumor effect of DOX [12]. Paclitaxel is used as an anticancer treatment, but it comes with challenges such as severe allergic reactions, multidrug resistance (MDR), and limited ability to cross the intestinal barrier. However, nanomicelles loaded with paclitaxel in TPH have shown outstanding physical characteristics and effectively suppressed the growth of A549/ADR cells. Once these nanomicelles enter acidic lysosomes, the degradation of HA by hyaluronidase leads to the formation of positively charged nanomicelles, which effectively induce mitochondrial OMM permeabilization by suppressing the antiapoptotic protein Bcl-2. This triggers the release of cytochrome C and initiates the activation of Caspases 3 and 9, ultimately leading to apoptosis [69, 70]. Betulinic acid (BA) has low absorption and poor solubility, but it demonstrates potent anticancer effects. To enhance its anticancer properties, a graft copolymer called Soluplus was used to encapsulate BA micelle (Soluplus-BA), synthesized using polyvinyl acetate, polyvinyl caprolactam, and polyethylene glycol (PVCL–PVA–PEG). According to the study, Soluplus-BA micelles remarkably amplified the inhibitory effects of BA on breast tumor cells. This was primarily due to their ability to induce increased accumulation of ROS and the disturbance of MMP [71]. The anticancer effect of micelles containing baicalein, D-*α*-tocopherol PEG 1000 succinate (TPGS), and pluronic F127 (F127) was thoroughly investigated. The baicalein micelles demonstrated a significantly higher potential to induce apoptosis compared to free baicalein. Moreover, the cell cycle study results conclusively demonstrated the ability of these micelles to effectively arrest cells at the G0/G1 phase. Notably, these micelles exhibited markedly improved solubility and enhanced oral bioavailability. It is evident that baicalein micelles effectively impede cell proliferation by utilizing the ROS-dependent mitochondrial-mediated apoptosis pathway [72]. The clinical use of triptolide (TPL) has been limited due to its toxicity and inefficient delivery. To address this challenge, Luo et al. developed a specialized thermosensitive hydrogel designed to release TPL in a controlled and localized manner within a tumor. The TPL@nano-gel exhibited increased cytotoxicity compared to the free form of TPL, attributed to its enhanced proapoptosis effect and regulation of endogenous mitochondrial pathways in BC cells. Furthermore, the TPL@nano-gel demonstrated a notable ability to inhibit angiogenesis by targeting VEGFR-2 signaling, highlighting its potential for further research [73]. A recent study has introduced a mitochondria-targeting camptothecin (CPT) polyprodrug system (MCPS). These micelles demonstrate exceptional stability and significantly enhance drug accumulation in tumor cells and tissues. Notably, the prodrug system incorporates triphenylphosphonium bromide, which effectively depolarizes the MMP, facilitating the transport of CPT into the mitochondria. Additionally, the intracellular reductant glutathione can cleave the disulfide bond in MCPS, leading to a substantial increase in mitochondrial DNA damage and consequently inducing cell apoptosis [14]. The development of PDPA/TPGS@DOX pH-responsive micelles represents a significant advancement in addressing DOX resistance in BC. These micelles offer a promising solution by releasing DOX in response to the acidic pH in early endosomes after cellular uptake, effectively overcoming resistance. Moreover, the TPGS component plays a crucial role in enhancing the effectiveness of DOX by targeting mitochondria and reducing MTP. The remarkable sixfold reduction in the IC50 of DOX by PDPA/TPGS micelles indicates their potential for improving treatment outcomes. Additionally, the observed more efficient tumor growth inhibition (TGI) with PDPA/TPGS@DOX compared to free DOX highlights the constructive impact of this approach [15]. The development of a pH-sensitive copolymer (POT) by modifying pluronic with *α*-TOS using an acid-labile ortho ester (OE) linkage has shown promising results in combating MDR. In vitro cell experiments have demonstrated the significant potential of POT micelles to effectively counteract MDR by inhibiting drug efflux mediated by pluronic and inducing higher production of ROS through *α*-TOS. This leads to heightened cytotoxicity and pronounced initiation of apoptosis in MDR cells. Furthermore, the use of POT-DOX micelles loaded with DOX has exhibited remarkable drug accumulation and potent inhibition of tumor growth [34]. Additionally, TSD-30-F and TSD-34-F micelles have shown significant induction of apoptosis and enhanced antitumor activity in tested BC cells by regulating specific genes and proteins [78]. Ferroptosis, a regulated cell death mechanism triggered by lipid hydroperoxide accumulation and dependent on iron, presents an opportunity to overcome MDR in BC. A recent study has developed a hybrid polymeric micelle designed to effectively deliver DOX and reverse MDR in vivo. This innovative micelle combines pluronic F127, known for its long-circulating properties in the blood, with phenylboronic ester-grafted pluronic P123 (PHE), serving as an effective efflux and detoxification regulator. The micelles have shown enhanced capability in inducing apoptosis, possibly attributed to their ability to not only increase intracellular drug concentration by inhibiting P-glycoprotein (P-gp) mediated drug efflux but also to stimulate ROS production by reducing glutathione levels. Moreover, in vivo evaluation has demonstrated that F127/PHE-DOX not only efficiently accumulates at tumor sites, but also exhibits the most potent inhibition of tumor growth, resulting in an impressive 87.87% TGI. Importantly, this remarkable antitumor effect was accompanied by minimal side effects [79]. To address MDR, another study has developed mixed micelles combined with quercetin (QU) and doxorubicin [77]. These micelles have the potential to enhance cytotoxicity by increasing the intracellular concentration of DOX, and promoting DOX-induced apoptosis. Additionally, they reduce the expression of P-gp, leading to a decrease in DOX efflux, and trigger mitochondria-dependent apoptotic pathways, thereby enhancing the rate of DOX-induced apoptosis [80]. In a separate study, conferone (Conf) was used as an adjuvant alongside Dox to effectively induce apoptosis in BC cells. The administration of Dox-Conf-loaded micelles induces a significant increase in tumor cell apoptosis through the intrinsic pathway [39].

### 4.4. Dendrimer

Dendrimers, with their compact macromolecular structure ranging from 1 to 100 nm, exhibit exceptional precision and uniformity due to their three-dimensional and radially symmetric configuration [40]. Their ability to carry numerous functional groups enhances their overall functionality, making them highly suitable for chemical modifications. This unique characteristic allows dendrimers to acquire specific pharmacokinetic and biodistribution profiles, positioning them as promising and versatile platforms [42]. There are various types of dendrimers that have been successfully developed for drug delivery purposes, including PEG, poly(glycerol-co-succinic acid), poly(propylene imine) (PPI), polyamidoamine (PAMAM), poly(glycerol), citric acid-based variants, carbohydrate-based variants, and more. Among these, PAMAM and PPI dendrimers have been extensively researched and are considered the most promising vectors for medical applications [40]. Combining dendrimers with different anticancer drugs, like cisplatin and Dox, has been shown to significantly improve their efficacy in fighting cancer [43]. The application of PAMAM dendrimers has evolved to effectively target multidrug-resistant tumor cells. To address the uncertainty surrounding the transportation of nanoparticles in these cells, Ma et al. [71] expanded a promising approach. The innovative strategy focused on a glucose transporter-facilitated and MMP-2-activated mitochondrial-targeted conjugate. This conjugate, comprising a PAMAM dendrimer core comodified with TPP and PTX, demonstrated the ability to selectively target mitochondria and release PTX, resulting in substantial toxicity against MCF-7/ADR cells. This groundbreaking approach holds considerable promise for combatting multidrug resistance in tumor cells [46]. The combined utilization of pluronic F68 (PF68)–conjugated PAMAM dendrimer and DOX has shown substantial enhancement in the antitumor activity of the DOX-loaded conjugates. This enhancement has been extensively demonstrated in both in vitro and in vivo studies. These complexes have demonstrated an exceptional capacity to increase DOX accumulation through the utilization of caveolae-mediated endocytosis. Furthermore, the observed modulation of mitochondrial function and gene expression by these complexes has ultimately led to the induction of apoptosis [50]. A study was conducted to investigate the influence of PAMAM-G4-NH2 on the proliferation of MCF-7/ADR cells. The results of the study indicated that PAMAM-G4-NH2 exerted a dose-dependent inhibitory effect on the viability of BC cells. This antiproliferative effect was further observed to be due to G0/G1 phase cell cycle arrest and the induction of mitochondria-dependent apoptosis, both of which were in agreement with previous findings in sensitive tumor cells such as MCF-7 [51]. In a research effort, Matai, Sachdev, and Gopinath utilized Generation 5 polyamidoamine G5 PAMAM dendrimers to stabilize the surface of silver nanoparticles (DsAgNPs) and deliver the drug 5-FU with the aim of achieving a synergistic effect in cancer cells. The created 5-FU@DsAgNCs displayed a potent antiproliferative effect on lung and BC cells, as evidenced by the increased levels of ROS and the consequent induction of oxidative stress [52]. The researchers conducted a study aimed at enhancing the potential of cationic G5 PAMAM dendrimers as a method for drug delivery. This was achieved through the hydrophobic modification of the dendrimers by surface grafting with lipid-like myristic acid (My) tails. The objective of this modification was to improve the drug delivery capabilities of My-g-G5 domains by encapsulating tamoxifen (TAM), an estrogen agonist, within them. Additionally, in vitro drug release studies demonstrated that My-g-G5/TAM complexes are capable of gradually releasing TAM in acidic environments and exhibited strong inhibitory effects in BC cells [53]. The combination of chemotherapeutics with RNA interference (RNAi) offers a promising strategy for enhancing the efficacy of cancer treatment and potentially achieving cancer eradication. In a study addressing resistance to TAM in BC, researchers aimed to suppress a mitochondrial enzyme known as manganese superoxide dismutase (MnSOD). Their objective was to hinder the transformation of TAM-induced ROS into less harmful compounds. To achieve this, they investigated the use of TAM in conjunction with MnSOD siRNA-delivering nanoparticles on BC cells. These nanoparticles, consisting of a siRNA/PAMAM dendriplex core and an acid-degradable polyketal (PK) shell, effectively restored TAM-induced cellular apoptosis in vitro and significantly inhibited tumor growth in vivo in TAM-resistant BC cells [21]. The conducted research on the anticancer properties of different generations of PAMAM dendrimers (G4 and G6) and their surface chemistries on HER2-positive BC cell lines has yielded promising outcomes. The study data demonstrates that PAMAM dendrimers, especially cationic ones, effectively diminished cell viability in a dose-dependent manner and prompted a notable increase in cell apoptosis. Moreover, the analysis revealed an elevation in the expression of apoptotic markers including Bax, Caspases 3, 8, and 9, alongside a reduction in the expression of Bcl-2. Furthermore, molecular pathway analysis indicated that PAMAM dendrimers heightened the expression of JNK1/2/3 and suppressed the activity of ERK1/2, EGFR1 (HER1), and HER2. These findings suggest that PAMAM dendrimers hold promise for potential development as efficacious treatments for HER2-positive BC [26].  Table 2 offers a detailed examination of the benefits and disadvantages associated with various nanotechnological approaches in mitochondrial-targeted therapies for BC treatment, providing valuable insights for future research and development in this area.

## 5. Application of Mitochondrial-Targeted Therapies in Clinical Trials for BC

There has been a growing interest in the application of mitochondrial-targeted therapies in the treatment of BC, evident from several clinical trials.

• MitoTam: MitoTam, an innovative mitochondrial-targeted derivative of TAM, has produced encouraging outcomes in preclinical evaluations and early-phase clinical trials. Notably, a Phase I trial involving patients with advanced solid tumors revealed MitoTam's favorable safety profile alongside preliminary indications of antitumor efficacy [88].
• Mitochondrial DNA-targeted therapy: Recent investigations have begun to assess the therapeutic potential of mitochondrial DNA (mtDNA) targeting as a strategy to induce apoptosis in cancer cells. This approach focuses on disrupting mtDNA functionality, leading to the selective death of malignant cells. Although still in nascent stages, this strategy shows promise in developing targeted therapies for BC [89].
• Targeting mitochondrial metabolism: Several clinical studies have examined the role of drugs that inhibit mitochondrial metabolism as a treatment modality. Metformin, primarily recognized for its antidiabetic properties, targets Mitochondrial Complex I, resulting in the attenuation of cancer cell proliferation. Ongoing clinical trials are further evaluating the efficacy of metformin and other mitochondrial metabolism inhibitors specifically within BC treatment paradigms [90].
• Mitochondrial transplantation: Recent research has investigated mitochondrial transplantation as a method to enhance antitumor efficacy in BC cells. Data indicates that such transplantation reduces ROS levels and elevates catalase activity, ultimately leading to diminished tumor growth [91].

The investigation of mitochondrial-targeted therapies for BC has generated encouraging preliminary results from preclinical studies and early-phase clinical trials [92]. However, further research is critical to refine these therapeutic strategies, evaluate their long-term safety and efficacy, and pinpoint the optimal treatment protocols for patient populations [93]. Currently, no mitochondrial-targeted therapeutic agents have secured scientific approval after entering the clinical treatment phase.  Table 3 showcases an exciting array of mitochondrial-targeted therapies that harness the power of nanotechnology. This innovative approach highlights the potential of tiny advancements making a significant impact on treatment options.

## 6. Drawbacks Associated With Mitochondrial-Related Therapies in BC

### 6.1. Drug Resistance in Cancer Therapies

Cancer cells exhibit a remarkable capacity to develop drug resistance when exposed to therapies targeting mitochondrial functions. This resistance can arise through various mechanisms, including alterations in mitochondrial dynamics and the activation of alternative survival pathways that allow the cells to endure treatment. Consequently, the effectiveness of these mitochondrial-targeting therapies diminishes over time, as cancer cells adapt and counteract their effects. In response to this pressing challenge, researchers are actively devising novel therapeutic strategies aimed at simultaneously targeting multiple survival pathways within cancer cells [99]. This innovative approach intends to enhance the inhibition of cancer cell survival, providing a more robust defense against the emergence of resistance to any single pathway. Additionally, scientists are exploring combination therapies that not only focus on mitochondrial functions but also engage other critical cellular components, such as DNA replication mechanisms and protein synthesis machinery [100]. By employing this multifaceted strategy, the objective is to more effectively eradicate cancer cells while minimizing the likelihood of drug resistance development. This comprehensive approach signifies a significant advancement in the ongoing struggle against cancer, offering hope for improved treatment outcomes [101].

### 6.2. Off-Target Effects of Mitochondrial-Targeting Therapies

Therapies targeting mitochondrial functions have shown considerable effectiveness in combating cancer cells; however, they can inadvertently affect healthy cells, leading to a variety of adverse reactions. These reactions may include common symptoms such as nausea and fatigue, as well as more severe issues like organ damage and, in some cases, the development of secondary cancers. The severity and nature of these off-target effects are influenced by factors such as the specific drug used and the prescribed dosage. This variability can limit the applicability of these therapies in certain patient populations or require careful monitoring to mitigate potential negative consequences [89].

This concern is particularly significant for individuals with pre-existing health conditions or those who exhibit increased sensitivity to these treatments. In such vulnerable groups, the anticipated therapeutic benefits may not sufficiently outweigh the associated risks [83]. To address these challenges, researchers are actively developing innovative drug delivery systems designed to precisely target cancer cells, thus sparing healthy tissues and reducing the likelihood of unintended harmful effects. By specifically honing in on cancer cells, these advanced delivery mechanisms aim to enhance treatment efficacy while minimizing risks to patients' overall health [102].

### 6.3. Lack of Specificity in Mitochondrial-Targeting Drugs

A notable limitation of mitochondrial-targeting therapies is their lack of specificity, which can lead to potential damage to healthy cells. This nonspecificity can compromise the efficacy of these treatments and limit their use among certain patient demographics [102]. To address this challenge, researchers are focused on developing innovative drugs that demonstrate increased specificity for cancer cells. In addition, combination therapies that target multiple aspects of cancer cells are being explored to minimize collateral damage to healthy tissues [17]. Researchers are also investigating “smart” drug delivery systems, such as nanoparticles and liposomes, which are designed to direct therapeutics specifically to cancer cells, thereby reducing adverse effects on normal cells. Ongoing studies aim to clarify the differences between cancerous and healthy cells, with the goal of creating new therapeutic agents that selectively target neoplastic cells while preserving the integrity of healthy tissue. This research has the potential to lead to more effective and safer treatment options for cancer patients [103, 104].

## 7. Mitochondrial-Targeted Therapies in Combination With Other Treatment Modalities

Several studies have explored the potential synergistic effects of combining mitochondrial-targeted therapies with other treatment modalities for BC. These combination approaches have shown promise in both adjuvant and neoadjuvant settings:

### 7.1. Chemotherapy

Combining mitochondrial-targeted therapies with conventional chemotherapeutic agents can enhance treatment efficacy by targeting multiple cellular pathways simultaneously. For example, studies have demonstrated the potential benefits of coadministering doxorubicin with mitochondrial-targeted liposomes or peptides, resulting in increased apoptosis and reduced tumor growth [105, 106].

### 7.2. Monoclonal Antibodies (mABs)

The combination of mitochondrial-targeted therapies with mABs, such as trastuzumab or pertuzumab, can potentially enhance targeted delivery and efficacy in HER2-positive BC. Preclinical studies have demonstrated the feasibility and potential benefits of this approach [107, 108].

### 7.3. Hormone Therapy

For hormone receptor–positive BC, mitochondrial-targeted therapies can be combined with hormone therapies, such as TAM or aromatase inhibitors, to improve treatment outcomes. By simultaneously targeting mitochondrial function and hormone-dependent pathways, this approach may overcome resistance and improve patient responses [108, 109].

### 7.4. Immunotherapy

Emerging evidence suggests that mitochondrial targeting agents can modulate the tumor microenvironment and enhance the efficacy of immunotherapies, such as immune checkpoint inhibitors. Combination strategies involving mitochondrial-targeted therapies and immunotherapy may provide a novel approach to boost the immune response against BC cells [110, 111].

In summary, combining mitochondrial-targeted therapies with other treatment modalities holds great potential for improving BC treatment outcomes by addressing multiple cellular pathways and mechanisms of resistance. Further clinical studies are needed to evaluate the safety, efficacy, and optimal combinations of these approaches in both adjuvant and neoadjuvant settings [112, 113].

## 8. Conclusion

The involvement of mitochondria in various biological processes is well-documented, and their dysfunction has been associated with tumor formation and cancer progression. Consequently, compounds that target mitochondria hold promise for cancer treatment. However, due to the complexity of mitochondria and challenges related to their selective and safe targeting, innovative approaches such as mitochondrial-targeted nanoparticles have been developed to transport drug-loaded nanoparticles to the mitochondria of cancer cells. These approaches have shown potential in improving the localization and accumulation of nanoparticles within the mitochondria, leading to enhanced therapeutic outcomes. Nano-based drug delivery systems possess several unique features, including a larger surface area and enhanced permeability, making them a potential game-changer in the fight against cancer. These advanced systems exhibit tremendous promise in facilitating various anticancer strategies. Despite these promising advancements, challenges in manufacturing and scaling up nanomaterials persist. Mitochondria-targeted nanoparticles have shown potential in fighting cancer while minimizing damage to healthy tissues and enabling real-time monitoring of treatment response. However, it is important to acknowledge that utilizing these nanoparticles in clinical applications presents difficulties, such as the lack of specificity for tumor cells in cationic nanocarriers. The interactions of cationic carriers with normal cells can lead to membrane integrity issues, cell death, and increased toxicity due to the abundance of positive charges on their surfaces. Additionally, in blood circulation, these carriers can be attracted to negatively charged proteins, leading to functional issues such as leakage, damage, and reduced circulation time. As a result, the use of cationic carriers like cationic liposomes or dendrimers in animal studies has been limited due to their toxicity and associated challenges, despite numerous studies being conducted. Therefore, there is a need to discover more biocompatible mitochondria-targeting molecules through practical design. Recently developed charge-reversible nanoparticles used for mitochondrial targeting could serve as better alternatives. Simplifying the design and construction of nanosystems to mitigate negative impacts on the carriers themselves is a critical challenge during their clinical translation. Additionally, the accumulation of nanomaterial inside cancer cells and their eventual localization to the mitochondria is relatively low, posing a challenge to these approaches. Addressing this challenge would require a design that enhances the controllability of these nanosystems. These challenges provide opportunities for further innovation and development in the field of nanotechnology for targeted mitochondrial therapy. Leveraging the microenvironment of mitochondria for drug release and regulation can significantly enhance the effectiveness of these methods. Furthermore, the precise targeting of nanocarriers to specific locations within mitochondria through external activation, such as electromagnetic fields or ultrasound, presents an opportunity to improve therapeutic outcomes. Addressing the challenge of rapid blood elimination and developing strategies to increase the circulation of mitochondrial nanoparticles should be a focus of future studies in this area. Additionally, overcoming the degradation of nanoparticles by proteinases and the obstacles posed by endosomes in delivering nanocarriers into cells are critical for achieving successful cytoplasmic transport. While the utilization of mitochondrial targeting nanoparticles for cancer treatment shows great promise, there is a clear need for multiple improvements to ensure their appropriate and highly effective clinical use. These challenges present opportunities for further research and innovation in the field of targeted mitochondrial therapy.

## 9. Futures and Perspectives

The application of mitochondrial-targeted therapies in the treatment of BC holds great potential due to their ability to exploit the unique characteristics of cancer cell mitochondria. However, several challenges must be addressed to fully harness the benefits of this approach.

### 9.1. Challenges

#### 9.1.1. Off-Target Effects and Toxicity

Mitochondrial targeting may also affect healthy cells, leading to undesired side effects. Further research is needed to enhance the specificity of these therapies towards cancer cells while minimizing harm to healthy tissue.

#### 9.1.2. Heterogeneity of BC Subtypes

The complex nature of BC necessitates the development of personalized mitochondrial-targeted therapies that can effectively target the specific molecular characteristics of different subtypes.

#### 9.1.3. Delivery and Pharmacokinetics

Ensuring efficient and targeted delivery of therapeutic agents to mitochondria within BC cells remains a challenge. Improving the pharmacokinetics, stability, and bioavailability of these therapies is crucial to enhance their efficacy.

#### 9.1.4. Drug Resistance

As with many cancer treatments, the development of resistance to mitochondrial-targeted therapies is a significant challenge. Overcoming resistance mechanisms and identifying synergistic combination therapies are essential for long-term treatment success.

### 9.2. Future Directions

#### 9.2.1. Personalized Medicine

Advances in genomic and proteomic profiling can enable the identification of specific mitochondrial vulnerabilities in individual patients, leading to personalized therapeutic strategies.

#### 9.2.2. Nanotechnology Advancements

Continued innovation in nanotechnology, such as the development of stimuli-responsive and actively targeted drug delivery systems, can improve the efficiency and specificity of mitochondrial-targeted therapies.

#### 9.2.3. Combination Therapies

The combination of mitochondrial-targeted therapies with other treatment modalities, such as immunotherapy, chemotherapy, and radiotherapy, may enhance treatment efficacy and reduce the development of resistance.

#### 9.2.4. Understanding Mitochondrial Biology

Continued research into the complex biology of mitochondria and their role in cancer will lead to the identification of novel therapeutic targets and approaches.

By addressing these challenges and pursuing these future directions, mitochondrial-targeted therapies hold significant promise for improving BC treatment outcomes and patient quality of life. Further multidisciplinary collaboration and translational research efforts will be crucial in realizing the full potential of this exciting field.

## Figures and Tables

**Table 1:** Nanotherapeutic approaches.

**Nano approach**	**Cell line/xenograft model**	**Conjugated/loaded molecule**	**Result**	**Date**	**Reference**
Lipophilic cation	MDA-MB-435	TPP-Dox	Significantly higher uptake, overcame the drug resistance in the cell line, and limited solubility	May 2014	[38]
	MDA-MB-468	[Au(d2pypp)2]Cl	Decreases mitochondrial membrane potential, depletes glutathione pools, activates Caspase 3 and Caspase 9, and induces apoptosis	October 2007	[39]
	MCF7, AU565, BT-549, MDA-MB-361, MDA-MB-231, and MCF 10F	Five TPP^+^-linked decyl polyhydroxybenzoates	Their mild uncoupling effect on OXPHOS triggers the mechanism responsible for cytotoxicity while also contributing to a reduction in mitochondrial transmembrane potential	October 2016	[40]
	MCF-7	Four meso-tetraphenylporphyrin derivatives	P1–P4 all target mitochondria. The results revealed that P1–P4 exhibited a level of phototoxicity towards the breast cancer cell line while having minimal dark toxicity at the concentrations tested	February 2010	[41]
	MDA-MB-468, 4T1, MDA-MB-231, and HCC1937	AuPhos-19 bearing a chiral phosphine ligand	The effective inhibition of the respiration process in mitochondria by AuPhos-19 is accompanied by the activation of AMPK. This leads to the depolarization of the mitochondrial membrane, the accumulation of ROS in the mitochondria, and the depletion of mitochondrial DNA	May 2022	[42]
	MDA-MB-231, MDA-MB-468, and HMEC	Gold(I) N-heterocyclic carbene complexes (Au(I) NHC)	Accumulate in mitochondria and induce apoptosis while also inhibiting the activity of thioredoxin reductase	August 2008	[43]

Liposome	Hela, 4T1 cell lines, and BALB/c mice	PTX-loaded TPP-PEG-PE	Targeting the mitochondria in cancer cells proved to be successful, leading to the efficient eradication of cancer cells in laboratory conditions and the inhibition of tumor growth in living organisms, as compared to using nontargeted PL loaded with PTX	May 2012	[44]
	Mice bearing 4T1 mammary tumors	DLP	The reported DLP platform successfully delivers combined photothermal and photodynamic therapies to mice with 4T1 mammary tumors, resulting in complete tumor eradication	April 2021	[45]
	MDA-MB-231	MI-PEOz-lip	Inhibiting the mitochondrial respiratory chain while also releasing IR780 to the tumor area	January 2023	[46]
	MDA-MB-231	PTN	Light and dose-dependent phototoxicity, accumulated both in mitochondria and lysosomes	March 2022	[47]
	MCF-7	PEGylated liposome and bioactive compounds from *Kappaphycus alvarezii*	The significant increase in ROS levels contributes to the damage of mitochondrial transmembrane potential	February 2020	[6]
	MCF-7	BTP	The increased production of ROS induces mitochondrial permeability transition, enhances the permeability of the mitochondrial membrane, and activates Caspase 3 and Caspase 7	January 2017	[7]
	MCF/ADR cells	DOX/CEL-MTS-R8H3	Enhanced mitochondrial targeting and effectively produced ROS	September 2022	[8]
	MCF-7, MDA-MB-435S	TPGS1000-TPP and sunitinib and vinorelbine	Immediate cytotoxic effect and triggered programmed cell death	September 2015	[48]
	Breast cancer orthotopic implanted mice	TPP-SS-ATS-LS	The enhanced cytotoxicity notably increased the inhibition rate of tumor growth from 37.7% to 56.4%. This was accompanied by the manifestation of mitochondrial dysfunction, decreased ATP production, and reduced respiratory capacity	August 2022	[9]
	TUBO cells	SS-02-Doxil	The enhancement of cytotoxicity, cellular binding, and uptake in TUBO tumor cells represents a significant improvement. Furthermore, there was a substantial increase in Caspases 3 and 9 activity, indicating a positive and constructive outcome	April 2021	[49]
	MDA-MB-231	KLA-5-FU/PTX	Amplified cytotoxicity, enhanced drug transportation to mitochondria, and triggered apoptosis	July 2022	[10]
	MCF-7 cells	Berberine	Inhibition of ABC transporters allows for the specific accumulation of the substance in the mitochondria. This leads to the activation of Bax, suppression of Bcl-2, release of cytochrome C, and subsequent activation of Caspases 3 and 9 enzymes	June 2013	[50]
	MCF-7/adr, MCF-7, and B16 melanoma	Topotecan	The substance is specifically directed to the mitochondria, leading to apoptosis, release of cytochrome C, and activation of Caspases 3 and 9 enzymes	February 2012	[51]
	MCF-7 and MCF-7/ADR	Epirubicin	Activated various apoptotic enzymes, including Caspases 3, 8, and 9. Upregulated Bax and downregulated Mcl-1 induced the generation of ROS through a cascade of events leading to apoptosis	2017	[52]
	MCF-7/MDR	Paclitaxel and lonidamine	Reduced intracellular ATP generation, sensitized multidrug-resistant breast cancer cells leading to apoptosis induction	December 2015	[53]
	MCF-7	Daunorubicin and quinacrine	The activation of Bax resulted in the dissipation of MMP, increased permeability of the mitochondria, release of cytochrome C, and triggered a cascade of Caspases 3 and 9 reactions, ultimately leading to apoptosis	January 2012	[54]
	MDA-MB-435S, MCF-7, and MDA-MB-231	Dihydroartemisinin and epirubicin	Effectively suppresses the function of Bcl-2, promotes the release of Beclin-1, and leads to the activation of Bax, triggered apoptosis, extended duration of drug circulation	October 2018	[11]

Micelle	MCF-7	Docetaxel and CGKRK_D_(KLAKLAK)_2_	The CGKRK peptide efficiently facilitates the transportation of the D(KLAKLAK)2 molecule to the mitochondria, effectively initiating mitochondria-dependent apoptosis. Moreover, docetaxel exerts an influence on microtubulin, resulting in the arrest of the cancer cell cycle	February 2017	[55]
	MDA-MB-231	PEG-IR780@Ce6	Improved absorption by tumor cells; generates ^1^O_2_, H_2_O_2_, and OH-free radicals; and suppresses migration and invasion	May 2021	[56]
	Mouse BC cell lines (4T1) and endothelial cells (EC)	DOX and EVO	EVO effectively damaged the mitochondrial membrane and DOX enhanced the antitumor effect, and overall inhibition of tumor growth	November 2019	[12]
	A549/ADR	TPH/PTX	Induced MOMP by suppressing Bcl-2. Activating apoptosis	January 2020	[57]
	MCF-7 and MDA-MB-231	DM-PEG-PCL NPs	Significant increase in mitochondrial damage, intensified expression of Caspase 3, and a decrease in Bcl-2, substantial reduction in tumor growth	November 2022	[58]
	MCF-7	PEG-IR780-BIIB021	Reduce the heat tolerance of cells, decrease the MMP, and activate intrinsic apoptosis factors	March 2021	[59]
	MDA-MB-231	PVCL–PVA–PEG encapsulated BA micelle (Soluplus-BA)	The enhanced accumulation of ROS and the disruption of MMP resulted in an increased inhibitory effect of BA. This, in turn, contributed to the improved antitumor effect of Soluplus-BA and its inhibitory effect on angiogenesis	September 2021	[60]
	MDA-MB-231	Baicalein, D-*α*-tocopherol polyethylene glycol 1000 succinate (TPGS), and pluronic F127 (F127)	Cell arrest at the G0/G1 phase, improved solubility, and enhanced oral bioavailability. Hinders cell proliferation by utilizing the ROS-dependent mitochondrial-mediated apoptosis pathway	December 2021	[61]
	MDA-MB-231 and MCF-7	TPL@nano-gel	Increased cytotoxicity, enhancing the proapoptosis effect, and inhibits angiogenesis through its inhibition of VEGFR-2 signaling	November 2020	[62]
	MCF-7, 4T1, and HeLa	Camptothecin (CPT) polyprodrug system (MCPS) and triphenylphosphonium bromide	High stability and enhanced accumulation of drugs in tumor cells depolarization of the MMP boost the destruction of mitochondria DNA, which consequently leads to the induction of cell apoptosis	July 2019	[14]
	MCF-7, 4T1, and MDA-MB-231	CSO-SS-Cy7-Hex/SPION/Srfn	Srfn administration through liposomal delivery resulted in significantly enhanced Srfn circulation time, which consequently prolonged the treatment duration of epithelial cancers, ultimately improving the overall therapeutic efficacy	August 2019	[63]
	MCF-7/ADR	PDPA/TPGS@DOX	The addition of TPGS to DOX has been found to significantly improve the efficacy of DOX by specifically targeting mitochondria, thereby reducing MTP and consequently lowering the IC50 of DOX by a remarkable sixfold. This results in a pronounced inhibition of tumor growth, enhancing the overall therapeutic efficacy	March 2015	[15]
	MCF-7 and MCF-7/ADR	POT-DOX	The synergistic use of ETC and PTT greatly increased the cytotoxicity, promoted apoptosis in MDR cells, and significantly augmented drug accumulation (3.03% ID/g/24 h), consequently leading to a potent inhibition of tumor growth (83.48%; TGI)	April 2020	[64]
	MCF-7, MDA-MB-231, and BALB/c mice	TSD-30-F and TSD-34-F	The synergistic use of ETC and PTT led to the remarkable upregulation of proapoptotic genes, such as Bax p53, Bak, and Caspase 3, while effectively downregulating the antiapoptotic gene Bcl-2, ultimately promoting cell death and inhibiting tumor growth	November 2022	[65]
	MCF-7, MCF-7/Adr, Caco-2, and gut sacs	Paclitaxel	The solubility of paclitaxel has been enhanced, enabling it to effectively combat resistant breast cancer. Moreover, the transport of paclitaxel has been improved, leading to better antitumor effects when orally administered to mice with xenografted-resistant breast cancers	April 2011	[66]
	MDA-MB-231	Dox-Conf	Significant increase in tumor cell apoptosis through the intrinsic pathway	November 2021	[67]
	MDA-MB-231/MDR1-bearing nude mice	Quercetin and DOX	Boosted cytotoxicity, triggered mitochondria-dependent apoptotic pathways, enhancing the rate of DOX-induced apoptosis	March 2020	[68]
	MCF-7/ADR	F127/PHE-DOX	Enhanced cytotoxicity, induced apoptosis, reducing GSH levels leading to increased ROS generation and 87.87% tumor growth inhibition	April 2020	[69]
	MCF-7 and MDA-MB-231	OXPt@Ch/VES	Made DNA damage, G2/M phase arrest, induced apoptosis, reduced nephrotoxicity, and prolonged survival	April 2022	[70]

Dendrimer	MCF-7/ADR	PAMAM and TPP and PTX	The increased tumor accumulation of the conjugate led to a more effective inhibition of tumor growth, minimizing the loss of body weight in the mice. This improved efficiency was achieved while simultaneously reducing the toxicity of the treatment, ultimately resulting in an overall enhanced therapeutic efficacy	March 2018	[71]
	MCF-7/ADR	PAMAM-PF68 and DOX	Increased accumulation of DOX by using caveolae-mediated endocytosis, modulated mitochondrial function and gene expression, induced apoptosis	November 2016	[72]
	Jimt-1	MitoTGFP-AuNPs and cationic maltotriose-modified poly(propylene imine)	Partial disruption of the outer mitochondrial membrane initiated a cascade of events, culminating in the induction of apoptotic signaling pathways, ultimately resulting in the programmed cell death of cancer cells	May 2015	[73]
	A549 and MCF-7	FU@DsAg	Significant increase in ROS levels and activates a gene cascade involving p53-mediated caspase signaling	March 2015	[74]
	MCF-7 and MCF-7/ADR	PAMAM-G4-NH2	A dose-dependent reduction in cell viability was observed in breast cancer cells, attributed to cell cycle arrest at the G0/G1 phase and the subsequent induction of apoptosis via the mitochondria-dependent pathway, indicating a potent anticancer therapeutic effect	March 2023	[75]
	MCF-7	My-*g*-G5/TAM	The treatment demonstrated strong inhibitory effects on cancer cell proliferation by elevating reactive oxygen species (ROS) levels, altering mitochondrial membrane potential (MMP), and ultimately exerting potent apoptosis-inducing abilities, resulting in a significant anticancer therapeutic effect	February 2016	[76]
	MCF7-BK-T	MnSOD siRNA/PAMAM-PK-TAM	Induced apoptosis and suppressed tumor growth	December 2013	[21]
	MCF-7 and MDA-MB-231	PtMet2–PAMAM and imidazole platinum(II)	The treatment was found to induce apoptosis through the activation of Caspase 9 and Caspase 8, which in turn stimulated the production of reactive oxygen species (ROS) via peroxidase (POX) activation. In parallel, p38 pathway activation led to an increase in Beclin-1 and LC3 expression, triggering autophagy. Simultaneously, the inhibition of AMPK and mTOR, the key regulators of autophagy, further enhanced the anticancer efficacy, showcasing a comprehensive therapeutic approach	May 2021	[20]
	SKBR3 and ZR75	Naked PAMAM	The treatment caused a marked decrease in cell viability, leading to the induction of apoptosis through the upregulation of proapoptotic proteins Bax; Caspases 3, 8, and 9; and the corresponding downregulation of the antiapoptotic protein Bcl-2. Additionally, the treatment enhanced the expression of JNK1/2/3 while suppressing ERK1/2, along with the activities of the epidermal growth factor receptor (EGFR1 and HER1) and human epidermal growth factor receptor 2 (HER2), demonstrating its comprehensive anticancer effects	May 2021	[26]

**Table 2:** The benefits and disadvantages of various nanotechnological approaches in mitochondrial-targeted therapies for breast cancer treatment.

**Nanotechnological approach**	**Benefits**	**Disadvantages**	**References**
Lipophilic cation	- Efficient delivery across membranes - Targeting mitochondrial membrane potential - Enhanced drug accumulation in cancer cells	- Nonspecific interactions - Cytotoxicity in healthy cells - Limited applications	[81, 82]

Liposome	- Improved drug solubility, stability, and bioavailability - Reduced systemic toxicity - Biocompatibility and biodegradability	- Complex formulation process - Instability in certain environments - Rapid clearance by RES	[83, 84]

Micelle	- Enhanced drug solubility - EPR effect in tumor tissues - Tunable properties	- Limited drug-loading capacity - Premature drug release - Potential toxicity and immunogenicity	[83, 85]

Dendrimer	- High drug-loading capacity - Controlled release and targeted delivery - Functionalization with targeting ligands or imaging agents	- Synthetic complexity - Cytotoxicity associated with cationic dendrimers - Potential immunogenicity and nonspecific interactions	[86, 87]

**Table 3:** Overview of investigating mitochondrial-targeted therapies utilizing nanotechnology.

**Drug/nanoparticle**	**Targeted mitochondrial pathway/function**	**Reference**
Doxorubicin/nanoparticle albumin-bound (nab) paclitaxel	Mitochondrial damage and apoptosis	[94, 95]
Mitochondria-targeted drug delivery system (DDS) with paclitaxel	Targeted drug delivery to mitochondria	[96, 97]
Nanoliposomal curcumin	ROS generation and mitochondrial depolarization	[98]

## Data Availability

The data utilized in this research can be accessed upon request, ensuring transparency and collaboration.
